# Multiphysics and multiscale modelling, data–model fusion and integration of organ physiology in the clinic: ventricular cardiac mechanics

**DOI:** 10.1098/rsfs.2015.0083

**Published:** 2016-04-06

**Authors:** Radomir Chabiniok, Vicky Y. Wang, Myrianthi Hadjicharalambous, Liya Asner, Jack Lee, Maxime Sermesant, Ellen Kuhl, Alistair A. Young, Philippe Moireau, Martyn P. Nash, Dominique Chapelle, David A. Nordsletten

**Affiliations:** 1 Division of Imaging Sciences and Biomedical Engineering, King's College London, St Thomas’ Hospital, London SE1 7EH, UK; 2 Inria and Paris-Saclay University, Bâtiment Alan Turing, 1 rue Honoré d'Estienne d'Orves, Campus de l'Ecole Polytechnique, Palaiseau 91120, France; 3 Auckland Bioengineering Institute, University of Auckland, 70 Symonds Street, Auckland, New Zealand; 4 Department of Engineering Science, University of Auckland, 70 Symonds Street, Auckland, New Zealand; 5 Inria, Asclepios team, 2004 route des Lucioles BP 93, Sophia Antipolis Cedex 06902, France; 6 Departments of Mechanical Engineering, Bioengineering, and Cardiothoracic Surgery, Stanford University, 496 Lomita Mall, Durand 217, Stanford, CA 94306, USA

**Keywords:** cardiac mechanics, data–model fusion, heart mechanics, patient-specific modelling, translational cardiac modelling

## Abstract

With heart and cardiovascular diseases continually challenging healthcare systems worldwide, translating basic research on cardiac (patho)physiology into clinical care is essential. Exacerbating this already extensive challenge is the complexity of the heart, relying on its hierarchical structure and function to maintain cardiovascular flow. Computational modelling has been proposed and actively pursued as a tool for accelerating research and translation. Allowing exploration of the relationships between physics, multiscale mechanisms and function, computational modelling provides a platform for improving our understanding of the heart. Further integration of experimental and clinical data through data assimilation and parameter estimation techniques is bringing computational models closer to use in routine clinical practice. This article reviews developments in computational cardiac modelling and how their integration with medical imaging data is providing new pathways for translational cardiac modelling.

## 1. Introduction

**Figure 1.**
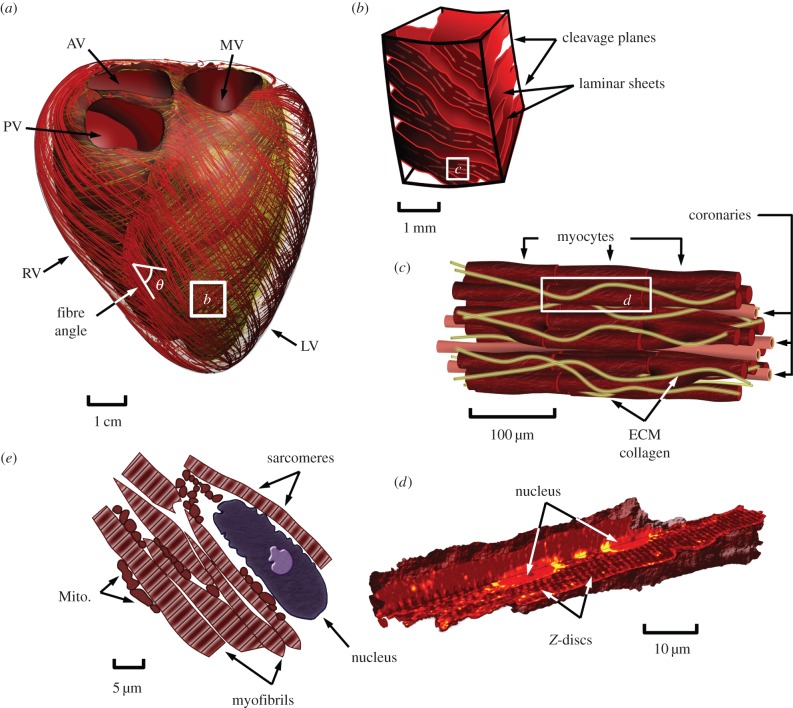
Illustrative representation of multiscale cardiac anatomy. (*a*) Geometric representation of the biventricular anatomy of the heart with streamlines illustrating its fibre architecture, (*b*) tissue block illustrating the laminar structure of the heart comprising fibre bundles arranged into sheets separated by cleavage planes, (*c*) local structural arrangement of myocytes and coronary capillaries, (*d*) 3D view of the cardiomyocyte cut to view internal structures (data courtesy of Dr Rajagopal and Dr Soeller [1,2]), (*e*) anatomy of the cell illustrating nucleus, myofibres (comprising crossbridges) and mitochondria. RV, right ventricle; LV, left ventricle; PV, pulmonary valve; AV, aortic valve; MV, mitral valve; ECM, extracellular matrix; Mito., mitochondria.

Heart function is the orchestration of multiple physical processes occurring across spatial scales that must act in concert to carry out its principal role: the transport of blood through the cardiovascular system. Interest in cardiac physiology stretches beyond scientific curiosity to genuine need, with diseases of the heart posing significant challenges to the vitality of societies, healthcare systems and economies worldwide. Discord in cardiac function leading to pathology can occur at every spatial scale (figure 1). Changes in protein isoforms in the contractile unit of the heart (sarcomere), in gene expression and organization of proteins, in the constitution of the extracellular tissue scaffold, in the flow of blood through the muscle, in the excitation of the muscle or in the anatomy of the organ highlight a few of many examples. The cumulative effect of pathology in heart disease—often an ensemble of multiple modifications—ultimately re-tunes cardiac function, leading to a progressive deterioration in performance as the heart struggles to maintain output. Cardiology has advanced significantly, improving care and outcomes in patients. However, our ability to diagnose specific mechanisms, plan or adapt therapy, and/or predict treatment outcomes in patients continues to present challenges, reflecting a need to improve how we use knowledge of cardiology to analyse—or model—the heart.

The use of modelling for understanding cardiac physiology and mechanics has a long history, with work stretching back to Woods [3], who estimated the stress in the heart wall by approximating the ventricle as a thin-walled spherical shell (known as the *Law of Laplace*). Modelling has since progressed to better approximate the underlying anatomical and physiological complexities. Geometric models of the heart evolved from thin-walled spheres to ellipsoids [4], axisymmetric idealized ventricles [5] and eventually to three-dimensional (3D) anatomically accurate geometries [6,7]. Studies illustrated the importance of accounting for nonlinearity in tissue mechanical properties [8–10] and structure [10,11] to understand the motion and load response of the heart. With experimental data on myocardial load response [12,13] and structure [14], more detailed structure-based models were introduced [15–17]. Experimental studies illustrating length [18], velocity [19] and frequency-dependent [20] modulation of muscle force were also integrated into models [21], broadening the scope of these models to simulate the mechanical function of the heart.

The biomechanical aspects of cardiac function cannot be fully isolated, instead they are interlinked with numerous physiological processes. At its core, the heart is a multiphysics organ [22] with electrical activation stimulating muscle contraction [23,24], muscle contraction interacting with intraventricular blood to promote outflow [25,26] and coronary perfusion [27], transporting metabolites and clearing waste products. These physical phenomena—involving reaction–diffusion, nonlinear mechanics, hemodynamics and biotransport—are tightly integrated, influencing the mechanical action of the heart. Heart function is dynamically regulated and modulated through interactions with the circulatory system, nervous system and endocrine system to change cardiac output [28–30]. Like other muscles, the heart's functional capacity and structure is also dynamic, responding to chronic changes in loading conditions and metabolic demand by altering cell structures, structural content, size, shape and vascular transport, among others. These physical and regulatory processes (which have been modelled to varying degrees) operate collectively to effectively deliver blood over decades and under extreme changes in functional demand. Understanding the influence of these factors on the biomechanics of the heart is important for deciphering the heterogeneous pathologies of the heart.

As the computational modelling community has explored various aspects of cardiac function at a fundamental level, parallel advancements in medical imaging and other diagnostic tools have enabled acquisition of an impressive range of datasets. Modern imaging is capable of recording the anatomy and motion of the heart, its tissue architecture, blood flow and perfusion, metabolism as well as numerous other pictures potentially useful in characterizing the state of a patient's heart [31]. With such a wealth of data, however, the challenge becomes integration and contextualization. Addressing this challenge is the primary aim of data assimilation, fusing cardiac models with these disparate data sources. While the complexity of a model is, in many ways, no simpler than the data it is founded upon, the advantage is its ability to link observed behaviour to the underlying physiology, providing potential mechanistic explanations of patient pathology. After the challenges of data–model fusion are met and model analysis provides novel insight, the difficulty then turns to translating these findings into clinically useable decision-making tools that can be robustly tested through clinical trials, proving their efficacy and superiority compared to existing techniques. This effort is the principle aim of *translational cardiac modelling* (TCM), bringing cardiac modelling and model-based outcomes into the clinical routine. TCM, however, remains in its infancy, grappling with the challenges of using real data and striking the balance between necessary and known complexity to deliver tools that address clinical needs. While the road to translation is challenging, the potential for significant impact has fuelled scientific progress forward towards this aim.

In this review, we consider the recent advances in ventricular cardiac mechanics modelling and translation to clinical applications. Section 2 provides an overview of the current state-of-the-art in biomechanical heart modelling, addressing the various aspects of multiphysics physiology explored in the literature. Emerging work in the areas of multiscale modelling as well as growth and remodelling applied to cardiac mechanics are also reviewed. Section 3 continues by reviewing available clinical data, the challenges in model parametrization as well as data–model fusion techniques commonly used in TCM. Finally, current translational efforts appearing in the literature are reviewed (§4) and future directions for TCM are discussed (§5). Exhaustive, in-depth review of each of these subjects could be an article in and of itself. Instead, this article gives an overview of the *road to translation* in cardiac mechanics, touching on the important aspects which span research disciplines and examining those future directions required to realize the potential impact of modelling. While this article focuses on translation of ventricular mechanics models in the heart, this discussion reviews tools and advancements that may facilitate other translational modelling efforts.

## 2. Modelling paradigms in the heart

The multiscale anatomy and physiology of the heart play an integral role to cardiac function [28] (figure 1). The cardiac muscle (or myocardium) is a highly structured collection of cells known as cardiomyocytes. Within these cells, the fundamental contractile unit is the sarcomere, consisting of inter-digitating lattices of thick and thin filaments. The cyclic interaction between the thick and thin filaments, triggered and regulated by intracellular calcium, determines the amount of force generated during active muscle contraction. Electrical excitation of the myocytes cyclically modulates intracellular calcium concentration, releasing calcium from internal stores that are subsequently replenished.

Myocytes are arranged axially through the extracellular matrix, providing the heart's necessary elastic structure. These fibres run in parallel and are separated by cleavage planes [14,32], interconnecting to form a 3D network allowing the heart to undergo complex motions during each cardiac cycle. The tissue organization enables fast propagation of electrical waves in the direction of fibres through gap junctions that interlink cells. The integrated electromechanical action of the heart is essential for the efficient transfer of blood through the cardiac chambers, coronary arteries and the entire circulatory system. The introduction of disease can occur through a variety of mechanisms, often leading to remodelling of the shape, structure and function of the heart as it aims to preserve output. However, long-term remodelling is often debilitative, leading to the progressive deterioration of cardiac performance and the development of heart disease. In this section, we review modelling efforts presented in the literature aimed at addressing the varying physiological aspects of the heart.

### 2.1. Modelling cardiac anatomy and structure

Personalized ventricular mechanics analyses rely on anatomically realistic geometric models. Since the early 1970s, sophisticated imaging techniques, such as cine-angiography and echocardiography (ECHO), have been used to provide detailed descriptions of the ventricular anatomy. Concurrently, experimental methods were designed that were capable of obtaining detailed 3D measurements of *ex vivo* cardiac geometry as well as myocardial microstructural information. This approach enabled the creation of high fidelity 3D anatomical models of dog [14,15], pig [33] and rabbit [34] hearts with embedded descriptions of the myocardial tissue architecture.

With the development of non-invasive 3D cardiac imaging techniques, the construction of subject-specific 3D anatomical models of the heart is now widespread. The availability of *in vivo* imaging has not only ensured that computational models can accurately represent the shape of intact hearts, but has also enabled *in vivo* mechanics analyses to be performed on a wide range of species (e.g. dog [35], sheep [36] and human [37–40]). Methods for constructing 3D anatomical models can be broadly categorized into three main approaches: (1) iterative nonlinear least-squares fitting [15,33,40–42], combining medical image registration with free-form deformation techniques to customize a generic mesh [43]; (2) volume mesh generation from binary masks or surface meshes of the myocardium performed by software packages such as *Tarantula* (http://www.meshing.at/), *GHS3D* (http://www-rocq.inria.fr/gamma/ghs3d/ghs.html), *CGAL* (http://www.cgal.org/) and *Simpleware* (http://www.simpleware.com/); and (3) cardiac atlases used for the construction of 3D models of the heart from non-invasive medical images [44,45]. While each approach has proved effective, limitations remain. Fitting techniques working from template meshes can struggle to preserve mesh quality and perform poorly when dissimilarity between the template and target geometry exists. In contrast, volume mesh generation from masks or surface segmentations usually have good mesh quality but are more sensitive to potential segmentation errors.

In addition to constructing anatomy, models must also incorporate myocardial tissue structure. Our basic understanding of 3D myocardial architecture has been built up from detailed histological studies [14,15,46]. These data are still widely used as the basis for rule-based algorithms to represent myocardial fibre orientation [47,48]. High spatial resolution data have been made available by scanning electron microscopy and confocal microscopy [14,32,41,42], and this has enabled quantitative characterization of myocardial laminae, which have been shown to play a significant role in cardiac mechanics [49,50]. Diffusion tensor imaging techniques have been developed to estimate myocyte orientations [51]. Recently, high spatial resolution images of *ex vivo* hearts have provided another means to study myofibre structure [52] and to examine the organization of laminae throughout the whole heart volume [53]. While progress towards more comprehensive methods for characterizing cardiac microstructure (particularly *in vivo*) are in development, lack of tissue structure information remains an important gap in computational models and a likely confounding factor influencing model results.

### 2.2. Passive myocardial constitutive equations

It is generally accepted that the mechanical response of ventricular myocardium can be described by anisotropic, hyperelastic (or viscoelastic) constitutive equations [12,54] (table 1). Transversely isotropic constitutive equations have been proposed by Humphrey & Yin [54] and Guccione *et al*. [60]—which has become one of the most cited and used cardiac constitutive models. The existence of myocardial sheetlets motivated the development of an orthotropic Fung-type exponential strain energy density function [74]. The model aimed to account for the relative shear (sliding) between adjacent sheetlets that have been shown to contribute to the total LV wall thickening during systole. Dokos *et al*. [66] confirmed that myocardium exhibits orthotropic mechanical response using carefully designed shear experiments on myocardial tissue blocks excised from the mid-ventricular wall. Similar material response was observed in shear testing experiment of human myocardium [75,76]. However, the level of statistical significance suggesting an orthotropic response was not as strong as that reported by Dokos *et al*. [66], with the mechanical response appearing transversely isotropic under large strain loads. Extending the approach pioneered for characterization of arteries [77,78], Holzapfel & Ogden [67] proposed a constitutive modelling framework, based on the underlying morphology of the heart tissue, to model the orthotropic passive mechanical response. Many constitutive equations in table 1 employ exponential forms, which generally suffer from a strong correlation between constitutive parameters. Criscione *et al*. [62] addressed these issues, in part, by using mutually independent strain invariants to define a transversely isotropic constitutive equation, minimizing the covariance between each of the response terms. However, this technique is yet to be used by the cardiac mechanics research community for predictive mechanics simulations.

**Table 1.** Table of sample of cardiac constitutive equations published and used in the literature. HE, hyperelastic; VE, viscoelastic; 1D, one-dimensional; ISO, isotropic; TISO, transversely isotropic; ORTH, orthotropic; UA, uni-axial; BA, bi-axial; MA, multi-axial; SH, shear; PV, pressure–volume; ES, epicardial strains; LitVals, various literature values.

model	type	structure	par no.	data	references	year
Humphrey & Yin	HE	TISO	4	BA [13]	[55]	1987
Horowitz	HE	TISO	8	BA [13]	[56]	1988
Humphrey	HE	TISO	5	BA [57]	[58]	1990
Guccione	HE	TISO	5	ES [59]	[60]	1991
Lin & Yin	HE	TISO	4	MA	[61]	1998
Criscione	HE	TISO	—	—	[62]	2001
Costa	HE	ORTH	7	—	[49]	2001
Pole-zero	HE	ORTH	18	LitVals, BA [63]	[17]	2001
Kerckhoffs^a^	HE	TISO	4	BA [57], PV [64]	[65]	2003
Holzapfel & Ogden	HE	ORTH	8	SH [66], BA [13]	[67]	2009
Loeffler	VE	1D	5	UA	[68]	1975
Yang	VE	ISO	5	UA	[69]	1991
Huyghe	VE	TISO	11	UA [70], BA [12]	[71]	1991
Holzapfel	VE	ORTH	4	LitVals	[72]	1991
Cansiz	VE	ORTH	17	SH [66]	[73]	2015

^a^Law also contains additional parameters for modelling tissue compressibility not included in the table.

Cardiac tissue comprises cells (principally myocytes and fibroblasts) surrounded by extracellular fluid and held together structurally through extracellular matrix proteins [79]. The tissue–fluid interaction gives rise to viscous effects, which have been experimentally demonstrated by the appearance of hysteresis in stress–strain [12] and force–displacement [66] curves during cyclic loading. Modelling the complex viscous effect poses challenges since the mechanical behaviour is time-dependent and the constituents of the computational models need to take into account a mixture of solids and fluids. Several methods have been proposed to represent viscoelastic function in passive constitutive models (table 1) ranging from relatively simple arrangements using Maxwell's constitutive equation [68] to more complex variants incorporating known orthotropic hyperelastic models [73]. Current viscoelastic models tend to provide capacity to appropriately model cyclic loading conditions. However, validation of these types of models demands further experiments specifically targeted for hysteresis and creep phenomena. These experiments can be challenging, particularly *ex vivo* where tissue viability and changes to the extracellular fluid environment can confound the recordings.

### 2.3. Active contraction constitutive equations

Sliding filament theory by Huxley [80] has formed the foundation of many modern myocyte contraction models. Some of the first models that used this theory were proposed by Wong [81]. Subsequent models have concentrated on relating the active fibre tension to muscle length, time following stimulus, the interaction between Ca^2+^ and troponin C [82], and the effects of sarcomere length on Ca^2+^ binding and tension [83]. Guccione & McCulloch [84] estimated the active fibre stress using a *deactivation* contraction model, which placed specific emphasis on the deactivating effect of shortening velocity, as well as the dependence on shortening history. This model formed the basis of a time-varying elastance model [16] that was dependent on sarcomere length and length-dependent calcium concentration.

Hunter *et al*. [21] proposed an empirical cellular mechanics model (the *HMT* model), which considered the passive mechanical properties of the tissue, the rapid binding of Ca^2+^ to troponin C and its tension-dependent release (which occur at a slower rate), the length-dependent tropomyosin movement, the availability of Ca^2+^ binding sites and the crossbridge tension development. This model was shown to reproduce the response to isotonic loading and dynamic sinusoidal loading experiments. Based on the HMT model, Niederer *et al*. [85] proposed a more detailed time-dependent model with descriptions of contractile stress by taking the dynamics of calcium binding into account. Both models used a fading memory model to compute the active tension resulting from crossbridge kinetics. The Rice contraction model [86] instead used phenomenological representations to approximate the spatial arrangement of crossbridges, creating a compact ordinary differential equation model capable of replicating a wide range of experiments. The Bestel–Clement–Sorine (BCS) contraction model (proposed in [87] and further developed in [88]) considered myosin molecular motors, was chemically controlled and consistent with the sliding filament theory. The passive component was designed to be transversely isotropic by combining an elastic model of the Z-discs with a viscohyperelastic component model representing the extracellular matrix. The active contractile component was also coupled with a viscous element. Transverse active stresses (orthogonal to the myocyte axis) have also been predicted by the arrangement of the myosin crossbridge [89] and applied in contraction models to achieve better matching of measured systolic strains [90].

In comparison to the *active stress* framework whereby passive and contractile stresses are summed, an *active strain* framework has also been proposed. Using concepts from the growth and remodelling literature, this approach defines the deformation gradient tensor as the product of a passive elastic component and an active component [91]. This approach aims to preserve convexity in the material strain energy by avoiding the stress superposition found in more traditional active contraction mechanics models. However, these theoretical results rely on the complete decoupling between active components and length-/velocity-dependent mechanisms, which may present challenges for the model's consistency when comparing to physiological experiments.

### 2.4. Incompressible versus nearly incompressible formulations

Myocardial models introduced in the literature vary in their treatment of myocardial mass and its conservation. Physiologically, this debate stems from the fact that experimental studies have shown intravascular blood flow may account for changes of 5–10% in ventricular wall volume during each cycle [92]. A common approach used in a number of models [17,49,93] is to approximate the tissue as incompressible using the so-called mixed *u*–*p* formulation [94], which neglects these minor variations in mass content. An equally common alternative considers the myocardium as compressible (or nearly incompressible), whereby myocardial volume is lost or gained as a function of the effective hydrostatic pressure based on a pressure–volume constitutive equation. This approach is commonly applied using a displacement only formulation with additional parameter(s) (i.e. bulk modulus and, potentially, others) which scale terms that penalize changes in volume [65,90,95]. An alternative employed in some cardiac mechanics studies is the perturbed Lagrangian approach, whereby pressure and displacement are solved with the pressure–volume constitutive relation given as the required constraint [73,96,97]. Numerical considerations for these models, in the context of cardiac mechanics, were discussed in [98]. Use of different models seems often motivated by numerical considerations, leaving an open question of which strategy is most applicable when modelling cardiac tissue.

### 2.5. Electromechanics

**Figure 2.**
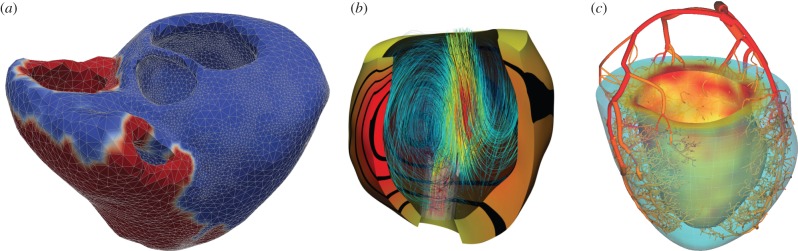
Samples of multiphysics modelling in the heart. (*a*) Biventricular electromechanical model of the heart illustrating the propagation of electrical potential over the heart [99]. (*b*) Fluid–solid mechanical model of the assisted LV. Fluid flow streamlines (coloured blue-red indicating increasing velocity magnitude) and myocardial displacements (yellow-red with equally spaced bands illustrating displacement magnitude) are illustrated [100,101]. (*c*) Coupled 1D flow-poroelastic perfusion model shown at early systole. Flow velocities are shown in the vessel segment. The pore pressure in the myocardium shows increased systolic compressive forces preferentially towards the subendocardium [102].

Contraction of the heart is induced by electrical activation initiating in the sinoatrial node and propagated through the myocardium by an electrophysiological depolarization wave. Coupling electrophysiology and mechanics allows models to account for known length-dependent alterations in wave propagation and resultant contraction. Electromechanical models (figure 2*a*) enable investigations of the role of abnormal electrical activity on mechanical performance of the heart as well as mechanical factors that contribute to arrhythmogenesis [24]. There are a wide variety of mathematical models of cardiac cell electrophysiology ranging from: low-order phenomenological models such as the FitzHugh–Nagumo (FHN) type models [103,104]; multivariate representations such as the Beeler–Reuter model [105] and biophysical ionic current models governed by ordinary differential equations [106], such as Hodgkin–Huxley type models [107] and human ventricular cell models [108]. The modified FHN model proposed in [104] has been widely adopted in electromechanics modelling studies [109–113], and species-specific models have been applied to study electromechanics during myocardial infarction [114] and dyssynchronous heart failure [115]. Mechano-electrical feedback plays an important role in the heart [116]. In particular, the state of deformation of the heart muscle is known to modulate the electrical properties of myocytes via the stretch-activated channels [113,117] and has the potential to modulate arrhythmogenesis. Although the electrical and mechanical function of the heart operate at significantly different timescales, coupled electromechanical simulations have been made possible via two numerical strategies. A common approach considers numerical partitioning of the electromechanical system, decoupling the electrical and mechanical problems using implicit–explicit methods [109,111,113,115,118,119]. Alternatively, a monolithic approach based on finite-element methods (FEMs) has been proposed [112], enabling stable integration of length-dependent effects of motion on electrical wave propagation. While quantitative comparisons between partitioned and monolithic approaches suggest the two forms tend to yield compatible results, these comparisons are likely to depend strongly on the selected model problem, with some responding more strongly to electromechanical coupling effects.

### 2.6. Fluid–structure interaction in the ventricles

Intraventricular blood flow and its influence on heart function has been the focus of numerous studies in the literature (see reviews by Khalafvand *et al*. [120] and Chan *et al*. [121]). Georgiadis *et al*. [122] did some of the first flow-specific work, considering the left ventricle as an axisymmetric ellipsoid. Similar models were later presented by Baccani *et al*. [123], Domenichini *et al*. [124] and Pedrizzetti & Domenichini [125], who suggested a possible link between ventricular vortical dynamics and disease. Saber *et al*. [126] and Merrifield *et al*. [127] presented some of the first patient-specific flow models of the LV using arbitrary Lagrangian–Eulerian (ALE) finite volume methods that integrated motion derived from images. Doenst *et al.* [128] and Oertel & Krittian [129] presented patient-specific flow models, incorporating left ventricle and atria, along with aortic structures for simulating left ventricular flow dynamics. Blood flow simulations have also been used to study diseases, such as myocardial infarction [130], congenital heart disease [131] and hypertrophic cardiomyopathy [132].

Some of the first models considering flow and tissue motion in the heart were done by Peskin [133], who focused on the interaction of flow with valves. This initial work spawned numerous subsequent studies examining the mechanical heart valves [134,135], mitral valves [136], both mitral and aortic valves [137] as well as recent work studying trileaflet biomechanical tissue valves [138,139]. Extension of fluid–structure interaction (FSI) techniques to study the interaction between blood flow and the ventricles was achieved by McQueen & Peskin [140,141], which was subsequently used for later studies of the heart [142–144]. These models, representing the myocardium as a collection of 1D fibres, were used to study coupling between flow and tissue along with the interaction between chambers of the heart [25,145–147]. One of the first attempts at modelling ventricular fluid–solid coupling using the FEM was presented by Chahboune & Crolet [148], where a two-dimensional (2D) model incorporating an anatomically based cross—section of the heart was used to analyse the effects of coupled flow and hemodynamics. Other 2D axisymmetric models were later used to study FSI effects under assist device support [149], in patients with DCM [150] and to examine the sensitivity of myocardial stiffness on clinical parameters [151].

Watanabe *et al*. [152,153] presented a 3D FEM model of an idealized left ventricle, incorporating myocardial biomechanics based on the work of Lin & Yin [61], representing the first work to incorporate state-of-the-art biomechanical models. This work used conforming low-order finite-elements to approximate the FSI problem, simplifying the intraventricular flow dynamics compared to comparable hemodynamics models. Cheng *et al*. [154] presented a partitioned passive filling model which coupled refined finite volume blood flow with a thin-walled isotropic hyperelastic wall model. Biventricular models were later developed which treated the heart as a passive Mooney–Rivlin material with an isotropic exponential term [155,156], driving systole and diastole through inflow/outflow boundary conditions. This model was later extended to incorporate an anisotropic term and emulate contraction by scaling passive material stiffness parameters with time [157]. A non-conforming monolithic FSI method and model [26,158] were used to simulate passive/active cardiac mechanics on patient-specific geometries using the Costa constitutive equation [49]. This model was later applied to study congenital heart diseases [159,160] and assisted left ventricles [100,101,161] (figure 2*b*). Krittian *et al*. [162] presented some of the first validation work, developing an experimental heart setup and modelled using both FSI and boundary-driven flow modelling, illustrating good qualitative agreement between data and model particularly for the boundary-driven flow model. Gao *et al*. [163] recently merged FEM models of the ventricle with the immersed boundary techniques of Peskin to enable use of current constitutive models within this framework.

While FSI models enable simulation of a multiphysics phenomenon at the core of cardiac function, such simulations often require substantial computational resources. Moreover, due to the low viscosity of blood and dominance of pressure, approximations of ventricular mechanics that ignore hemodynamics (considering the ventricle as a pressure-filled chamber) have proved effective in many modelling scenarios. While it is established that intraventricular pressure gradients are present in the ventricle, the spatial variation in pressure is often small relative to the absolute pressure scale. On the other hand, FSI models become increasingly important when studying specific pathologies, such as valve stenosis or hypoplastic left heart syndrome where significant increases in shear stress or decreases in ventricular filling pressures are observed, respectively.

### 2.7. Poromechanical modelling

The coupling of myocardial deformation and coronary blood flow (perfusion) has long been an area of interest motivated, in large part, by the significant influence of the crosstalk on their respective function (for review, see [164]). Coronary perfusion, essential for delivering metabolites to the myocardium, is functionally altered by muscle contraction with changes in muscle stiffness and deformation influencing the vascular resistance and compliance. From a mechanical modelling standpoint, one may conceptualize this multiphysics coupling within a classical FSI framework, explicitly resolving each vessel in the tissue [165]. However, this approach presents challenges given the anatomical complexity of the coronary vasculature [102] and significant variation in scale—from epicardial arteries (on the scale of millimetre) to coronary capillaries (on the scale of micrometre). An alternative is to consider flow below some spatial scale as a continuum, no longer resolved on the basis of individual vessels but as a continuous phenomenon present over the myocardial volume [166,167]. Two main families of this multiscale approach are classically used: *homogenization* and *mixture theory*.

*Homogenization* refers to a family of mathematical methods in which the contribution of smaller scale flows is simplified by seeking an asymptotic limit for the problem solution assuming a scale separation between microscopic and macroscopic domains. A typical underlying assumption is the presence of periodicity in the geometry and material properties of the microstructure, which can be described by the spatial repetition of a basic *unit cell* (often referred to as a *representative volume element* in mechanical literature). Various asymptotic methods can then be used to mathematically characterize the limit solution. The limit formulation generally takes the form of a coupled problem between variables at both macroscopic and microscopic scales. In certain restrictive situations, i.e. cases involving linear microscale problems with constant coefficients, the microscopic problem can be solved independently of the macroscopic one. More generally, the formulation requires an iterative solution process, whereby variables in the unit cell are calculated assuming a given macroscopic solution and resultant quantities are passed to the macroscopic problem. This increases the computational demand for practical problems significantly; however, this approach has been successfully explored for perfusion modelling [168,169].

In contrast, *mixture theory* is directly formulated at the macroscopic level assuming that a given number of distinct solid and fluid phases coexist and interact at each point in the continuum. This approach differs from the previous approach by directly embedding the functional effect of the unit cell into the governing poromechanical system through the use of constitutive equations governing the kinematic–kinetic interaction within (and between) phases. This approach originated in the pioneering work of Biot [170] and has been further enhanced and refined over the decades, using general descriptions of continuum mechanics to express the fundamental principles of conservation laws and thermodynamics [171–173]. More recently, the particular challenges present in the heart (e.g. large strains and rapid fluid flows) have been specifically addressed [174]. This leads to a coupled formulation that resembles ALE FSI [175], except that here the fluid and solid domains are the same and a volume-distributed coupling term is present [176].

When considering the application of these poromechanical approaches to the heart, experimental observations reveal a number of important additional features. Regarding the passive behaviour, cardiac tissue displays strongly anisotropic swelling and stiffening effects under perfusion pressure [177], similar to that of skeletal muscle [178]. The active behaviour of the heart is also seen to exhibit notable coupling effects—such as the well-known flow impediment phenomenon occurring during cardiac systole [179]—that modelling must incorporate. Perfusion is also highly compartmentalized, with flow in a region of tissue fed by a specific set of larger arteries [180]. To address this, the explicit solution of the flow in larger arteries has been coupled to a distal poromechanical tissue model yielding a multiscale representation that accounts for the distribution of inflow sites [102] (figure 2*c*). Additionally, different scales of the vasculature—exhibiting largely different flow characteristics—often inhabit the same volume, pointing to the need for so-called *multi-compartment* formulations [169,181] that allow for generations of vessels to be treated independently. The relationship between the explicit vascular structure and permeability and porosity must also be either quantified from high-resolution microvascular imaging [182,183] or from perfusion imaging [184].

### 2.8. Multiscale modelling strategies for contraction

The ejection of blood occurring with each beat of the heart depends on a cascade of functions spanning multiple scales. The power stroke of each myosin head (motion on the nanometre scale) aggregates through the hierarchical structure of the organ to produce each heart beat (motion at the centimetre scale). As a result, understanding the biochemistry of the heart and its influence on mechanical function requires use of multiscale, or micro-macro, approaches. Many multiscale modelling approaches, particularly in electrophysiology [185–187], have been introduced to enable the integration of biophysical models through the hierarchy illustrated in figure 1. Here, we focus our discussion particularly on multiscale modelling for contraction.

#### 2.8.1. Subcellular contraction modelling

A crossbridge in itself can be seen as a special chemical entity having internal mechanical variables (or degrees of freedom) pertaining to the actual geometric configuration. In this context, whether a crossbridge is in an attached or detached state implies a conformational change of these internal variables and a change in the inherent free energy [188], providing a thermodynamically consistent basis for modelling the complex interplay of chemical and mechanical phenomena at the sarcomere level. This framework is appropriate for analysing the Huxley models of muscle contraction, based on the so-called *sliding filament theory*. In [80], the crossbridge is seen as a system with one mechanical degree of freedom associated with a linear spring that is under tension as soon as the myosin head attaches to the actin filament, which collectively induces muscle contraction. This model was later refined in [189], including additional chemical states in the attached configuration that accounted for the so-called power-stroke phenomenon, a key concept in the attachment–detachment cycle described in [190] (see also [191]). An alternative approach was recently proposed in [192], where a purely mechanical model was substituted for the chemical states of the attached crossbridge, with 2 degrees of freedom corresponding to one bi-stable element in series with a linear spring.

#### 2.8.2. Multiscale/micro-macro approaches for contraction

Once the crossbridge behaviour has been modelled, the major challenge is to aggregate the behaviour of a single crossbridge over myofibrils, myocytes, myofibres to motion at the tissue and whole-organ scales (figure 1). Given the large number of crossbridges at the sarcomere level and above, it is quite natural to adopt a statistical description, as proposed in [80], by which the total active force/stress is given by the mean of the individual crossbridge forces with an appropriate scaling factor. This approach naturally leads to considering dynamical equations representing the evolution of the underlying probability density functions (PDFs). In order to mitigate the resulting computational costs in the simulation process, some approximations can be made by representing the PDF by a limited number of so-called moments [193]. In fact, under certain modelling assumptions, the moments equations are exact, and directly provide the active stresses themselves as solutions of a dynamical equation [88,194]. In any case, active stresses need to be eventually considered in conjunction with passive behaviour ingredients, resulting from various constituents other than sarcomeres at the cell level and outside of cells. Use of homogenization could be envisioned for this purpose, albeit it is more standard in mechanics to resort to rheological modelling, by which various components can be combined in an energy-consistent formalism, e.g. in the three-element muscle model proposed by Hill [195] and used in [88] to formulate a complete macroscopic cardiac tissue model.

An important consideration is the adequacy of such multiscale modelling approaches and whether they appropriately link cardiac function across scales. It has been demonstrated in [80] that a 1 degree of freedom muscle contraction model is adequate for representing important features of muscle physiology, such as the Hill force–velocity dependence. Furthermore, the extension of this model proposed in [88] was shown to correctly reproduce length-dependence effects namely, the so-called Frank–Starling mechanism and the scaled elastance properties evidenced by Suga *et al*. [196] and observed in experimental measurements [197]. Nevertheless, an important motivation for a more refined microscopic model resides in the shorter time scale effects observed in fast transients, such as under force or length clamp [189,198]. In addition, transient deactivation may be a critical determinant of mechanical heterogeneity in asynchronous activation [199].

### 2.9. Growth and remodelling

The form and function of the heart changes continuously–during development and aging or in response to training, disease and clinical intervention [200]. These phenomena are collectively referred to as growth and remodelling [201]. Specifically, the notion of growth is commonly related to changes in cardiac dimensions, whereas the notion of remodelling refers to changes in cardiac structure, composition or muscle fibre orientation [202]. Modelling the long-term, chronic response of the heart through growth and remodelling is challenging, and far from being completely understood.

Proposed more than two decades ago [203], the first mathematical model for growth within the framework of nonlinear continuum mechanics is now well established and widely used for various biological structures [204]. These models are based on the conceptual idea to decompose the deformation gradient into a reversible elastic and an irreversible growth part. Only the elastic part (a product of the deformation gradient and the inverse growth tensor) is used in the constitutive equations, which then enters the equilibrium formulation in the standard way [201]. The growth part, a second-order tensor, can be prescribed using constitutive equations relating the growth kinematics and growth kinetics, two equations that characterize how and why the system grows [205]. As an alternative to model growth at the continuum level, is the constrained mixture approach [206], based on the original mixture theory of Truesdell & Noll [207], where each of the continuum's constituents possesses its own reference configuration, physical properties and rate of turnover, but deforms as a whole with the continuum.

Cardiac growth is associated with a wide variety of pathologies, which can conceptually be classified into two categories [208]: concentric and eccentric hypertrophy. Concentric hypertrophy is associated with thickening of the ventricular walls, impaired filling and diastolic heart failure. Eccentric hypertrophy is associated with a dilation of the ventricles, reduced pump function and systolic heart failure [209]. While the causes for cardiac growth are generally multifactorial, concentric hypertrophy is commonly believed to be a consequence of pressure overload, while eccentric hypertrophy is thought to be related to volume overload [210]. Both conditions can affect the left and/or right side of the heart [211]. Left-sided overload triggers growth and remodelling of the left ventricle and may cause the symptoms of left heart failure (pulmonary oedema, reduced ejection fraction or cardiogenic shock). Right-sided overload triggers right ventricular growth and remodelling and may cause symptoms of right heart failure (compromised pulmonary flow, stagnation venous blood flow and poor LV preload). These different manifestations of cardiac growth and remodelling are chronic and can progressively worsen over time, ultimately proving fatal.

A variety of different models for cardiac growth have been proposed throughout the past decade [212]. Some simply prescribe a growth constitutive equation and study its impact on cardiac function [213] without feedback mechanisms based on the physiological status of the heart. Others allow growth to evolve naturally in response to pressure overload or volume overload [210]. While initial conceptual models used idealized ellipsoidal single ventricles [213] or bi-ventricular geometries [205], more recent approaches use personalized human heart geometries with all four chambers [209]. Using whole heart models allows us to explore clinically relevant secondary effects of growth and remodelling including papillary muscle dislocation, dilation of valve annuli with subsequent regurgitant flow and outflow obstruction [209]. Growth models may also explain residual stress which is known to be present in the myocardium [214,215].

Multiscale modelling holds the potential to link the clinical manifestation of cardiac growth to molecular and cellular level events and integrate data from different sources and scales. Studies of explanted failing human hearts revealed that the type of cardiac growth is directly linked to changes in cellular ultrastructure [216]: in concentric hypertrophy, individual cardiomyocytes thicken through the parallel addition of myofibrils; in eccentric hypertrophy, cardiomyocytes lengthen through the serial addition of sarcomeres [217]. Eventually, integrating these chronic alterations of cardiomyocyte structure and morphology into multiscale models of cardiac growth could help elucidate how heart failure progresses across the scales. It would also allow us to fuse data from various sources—clinical, histological, biochemical and genetic—to gain a comprehensive, holistic understanding of the mechanisms that drive disease progression.

## 3. Towards translation: data–model fusion

Models adapted to a patient's cardiac anatomy and function are being proposed for use in diagnostic medicine—providing new or improved biomarkers for indicating or stratifying disease—as well as predictive medicine—allowing for virtual testing of treatment both acutely and longitudinally. Underpinning this translational approach are the significant advancements made in medical imaging which are now capable of providing a wealth of information about the anatomy, structure and kinematics of the heart. While the concept of leveraging this information to define patient-specific models is straightforward, the execution of fusing images and models remains a key challenge in many TCM projects. This process depends critically on the type of data used, image processing of data into quantifiable terms and assimilation of this data within a model.

### 3.1. Clinical data and acquisition

Since the first X-ray image acquired in 1895, medical imaging data have grown to play a key role in patient diagnosis, treatment planning and follow-up in the clinic. This is thanks, in large part, to the advent of new modalities, reduced cost of imaging, prevalence of systems in clinics worldwide and substantial body of evidence from clinical trials highlighting the improvement in patient outcomes for specific treatments using image-derived quantities. The main non-invasive imaging techniques used in cardiology and applied in cardiac modelling are ECHO, computed tomography (CT) and magnetic resonance imaging (MRI) [31].

**Figure 3.**
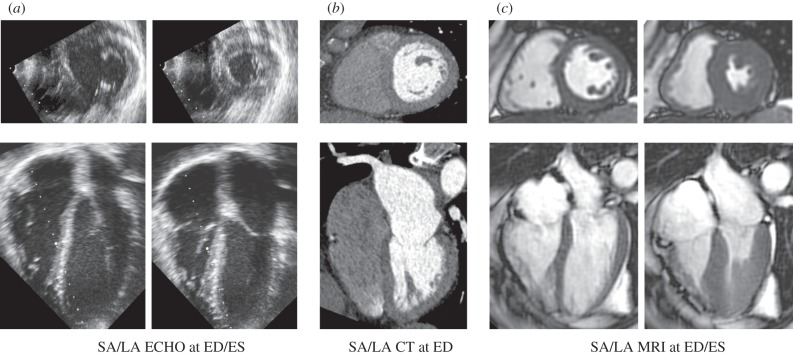
Example of typical medical images of short-axis and long-axis views of the heart. (*a*) ECHO images at two points in the cardiac cycle. (*b*) CT images at end diastole (single time point usually acquired due to radiation dose) with contrast bolus illuminating the LV blood pool. (*c*) CINE MRI at two points in the cardiac cycle. SA, short-axis; LA, long axis; ED, end diastole; ES, end systole.

Out of these three modalities, ECHO is by far the most accessible in clinics. It combines safety, low price and versatility for a wide range of cardiovascular disorders (assessment of anatomy and function of heart and valves, anatomy of large vessels, measurement of flow using Doppler effect and others). The excellent temporal resolution is compromised by a lower signal-to-noise ratio and contrast-to-noise ratio (figure 3*a*) and limitations in the reproducibility of exams, which are often operator-dependent. Although there are no real contraindications for the ECHO exam, the hearts of larger patients can be difficult to image. Regardless, the prevalence of ECHO in clinics worldwide and current use in assessment of heart conditions suggests that models capable of successfully exploiting this data source have a large potential for translational impact.

Current cardiac CT imaging (multi-slice and multi-source systems) has the advantage of fast acquisition, excellent spatial resolution and reproducibility (figure 3*b*). These factors enable morphological and functional assessment of the heart (including valves) even for larger patients. The superior spatial resolution of CT and fast acquisition times make it the modality of choice for the non-invasive assessment of detailed anatomy, such as stenosis in arteries or valves. CT can also provide dynamic information throughout the cardiac cycle, though this is usually avoided in patients due to the significant radiation dose. Indeed, the main drawbacks of CT are the patient exposure to ionizing radiation and application of iodine contrast agents. These drawbacks are, in particular cases, outweighed by the potential benefit of CT; for example, coronary angiography and other vascular stenoses as well as valve disorders.

MRI provides similar functional and morphological information as CT without the use of ionizing radiation (figure 3*c*). A higher temporal resolution (in comparison to CT) contributes to accurate functional imaging of heart [218,219]. In addition, MRI is capable of imaging tissue characteristics such as presence of infarction scar [220], fat infiltration or inflammation [221], fibrosis [222,223], iron-loading [224], 3D myocardial strains [225,226] and potentially vascular tree structure [227]. MRI is limited in the speed of acquisition, relying on cardiac gating, respiratory navigation or breath-holds to acquire images of the heart. As a result, typical MRI images are averages over multiple heartbeats. Speed of acquisition and signal-to-noise remain key factors that limit the spatio-temporal resolution of MRI. Advances in MRI acceleration techniques and improved motion correction, however, have led to substantial progress in imaging of fine anatomical structures (e.g. coronaries [228]). The main drawbacks of MRI are in contraindication for some patients (e.g. MRI incompatible pacemakers), longer acquisition times and the cost of exams.

While the spatio-temporal resolution of these modalities is often adapted for a given patient (e.g. breath-hold duration in MRI or X-ray dose in CT), table 2 provides some typical values. The details of image acquisition often play a significant role in later processing and present practical considerations important for translation. The lack of reproducibility in repetitive breath holding in MRI as well as differences in breath-hold and free breathing scans often lead to images that are not easily co-registered into the same orientation and space. Even in common short-axis cine MRI stacks, differences in a patient's breath-hold position can yield significant misalignment between 2D slices, an issue that is not problematic for clinical processing, but challenging for TCM. As a result, it is important to carefully adapt imaging protocols to optimize them when used in modelling.

**Table 2.** Sources of image data used in TCM. Resp.comp., respiratory compensation; RT, real time; PI(*n*), parallel imaging (acceleration factor); BH, breath-hold; FB, free breathing; NAV, breathing navigator; excl.NAVeff., excluding navigator efficiency (total scanning time needs to be multiplied 2–3×), kt, kt-blast/kt-sense/kt-PCA; NSA, number of signal averages; HB, heartbeat; PC MRI, phase contrast MRI. Note that the X-ray dose of non-dynamic CT is approximately 10-folds lower than in dynamic CT.

modality (MRI sequence)	acceleration (MRI)	resp. comp.	duration	resolution (mm) × (ms)
BH Cine MRI (bSSFP)	PI(2)	BH	5–8 s/slice	(2 × 2 × 8) × (20)
FB Cine MRI	PI(2)	FB(NSA)	60 s/slice	(2 × 2 × 8) × (20)
RT Cine MRI	PI(3–4)	FB	RT	(3.5 × 3.5 × 10) × (70)
3D morphology (bSSFP) [228,229]	PI(3)	FB, NAV	4 min excl.NAVeff	1.5 × 1.5 × 1.5
2D tagged [230]	PI(2)	BH	15 s/slice	(2 × 2 × 8) × (30)
3D tagged [225]	PI(2)	BH	15 s/volume × 3	(3 × 3 × 7) × (20)
T1-mapping (MOLLI)	—	BH	20 s/slice	2 × 2 × 8
MR perfusion (2D) [231]	kt(5)	BH/FB	3 slices/HB	(2.5 × 2.5 × 10) × (1000)
MR Perfusion (3D) [232]	kt(7)	BH/FB	volume/HB	(2.5 × 2.5 × 5) × (1000)
2D echo (RT)	—	FB	RT	(below 0.5 mm) × (20)
3D echo	—	BH	10 s	(below 1 mm) × (60)
CT (single time image)	—	BH	15 s	below 1 mm
CT (dynamic)	—	BH	15 s	(below 1 mm) × (70)
2D MRI flow	PI(2)	BH/FB(NSA)	15 s (in BH)	(2 × 2 × 8) × (20)
4D MRI flow [233]	kt(8)	FB	7 min excl.NAVeff	(2.3 × 2.3 × 2.3) × (30)
echo Doppler	—	FB	RT	(10 × 10 × 10)
*in vivo* DTI [234]	—	BH/FB	60 min/5 slices	(2.7 × 2.7 × 6)

Other advanced imaging methods in development could further enhance the data available for modelling. Dual positron emission tomography (PET) MRI or PET-CT are enabling the imaging of functions (such as metabolism and perfusion) in the heart along with co-registration to anatomical information. Elastography techniques using ECHO or MRI [235–237] introduce the potential of directly measuring apparent tissue stiffness at multiple points in the cardiac cycle [235]. Advancement of diffusion tensor imaging into the heart *in vivo* [234,238] provides the possibility to measure specific structural characteristics of the heart. While not yet used in the clinic, these modalities may play a role in future model-based parameter estimation.

Beyond imaging, a range of alternative data sources have proved useful in TCM. Pressure in the heart cavities (important for instance to obtain quantitative values of tissue stresses) can be measured by invasive catheterization. The non-invasive blood pressure measurement in periphery by using a transfer function [239] can provide the proximal aortic pressure over the cardiac cycle. Electrophysiology models can be constrained by invasively measured intracavity extracellular potentials, non-invasive body surface mapping procedures, as well as electrocardiogram recordings. The former was applied in CRT modelling [39,99] and the latter is a current challenge for a number of research groups.

### 3.2. Image processing

The use of acquired image data in developing patient-specific cardiac models is preceded by image processing. Often image processing is necessary for the personalization of model anatomy and the extraction of information from the data (e.g. motion pattern) that can be used in the personalization of the model (e.g. data assimilation).

As the information used in modelling is often combined using several types of data, spatial and temporal registration of the acquired images is necessary. Given the fast dynamics of a heart beat and the relatively small displacements involved, achieving a robust and accurate multi-modal registration of all the available data is core to the accuracy and robustness of model outputs. While within a single modality, the techniques of spatial registration are quite robust [240], the registration in between modalities often requires manual input/corrections.

An important step of image processing is image segmentation used to extract relevant anatomical details and extracting motion information, for instance displacement of points (out of 3D tagged MRI), motion of endo- and epicardial surfaces (cine MRI, 3D ECHO) or tag-plane motion (2D tagged MRI [241]). There are a number of existing segmentation and motion-tracking methods relying on manual, semi-automated or fully automated procedures. A move towards full automation is essential for wider use of personalized modelling techniques [242,243]. The uncertainty of image processing impacts the accuracy of the personalized model. Recent projects combining synthetic images and biophysical models proposed a way to estimate the accuracy of motion tracking in medical images [244].

Table 3 summarizes selected studies in which modelling was successfully combined with real or synthetic datasets. Typical image data modalities applied in these studies were cine or tagged MRI and myocardial passive and active properties were estimated. In some of the studies, parameters of an electrophysiological model (typically regional tissue conductivities) were estimated. Both *ex vivo* [255,256] and *in vivo* diffusion tensor imaging [238,257] data have been processed to estimate myocardial fibre orientations and used for simulations. Certain difficulties of using *ex vivo* images (including inter-subject registration, and the differences in tissue properties between excised and living organs) may be circumvented *in vivo* with the development of imaging [258] and reconstruction [48,238] techniques.

**Table 3.** Examples of works presented in the literature targeted at TCM and data–model fusion. CE, constitutive equation; est. meth., estimation methods; SA, sensitivity analysis; NH, neo-Hookean; GL, Guccione constitutive equation (law); NF, neo-Fibre; HO, Holzapfel–Ogden; V, variational; PS, parameter sweep; RO-UKF, reduced-order unscented Kalman filter; ML, manifold learning; DFO, derivative-free optimization; P, passive; A, active; BCS, Bestel–Clement–Sorine.

data	subject	CE	est. meth.	phase	SA	references
2D tagged	phantom	NH	V	P	—	[245]
2D tagged	phantom	GL	V	P	—	[245]
2D tagged	human	GL	V	P	—	[246]
3D tagged and cine MRI	human	GL	RO-UKF	P	—	[247]
cine MRI and pressure	human			A	—	[248]
2D tagged	*in silico*				—	[241]
cine MRI	pig	BCS	RO-UKF	P, A	X	[249]
cine MRI	human	BCS	UKF	P, A	X	[250]
cine MRI	human	BCS	ML	P, A	—	[251]
3D tagged and cine MRI	*in silico* and human	NH, NF, GL, HO	PS	P	X	[244,252]
cine MRI	human	BCS	DFO	A	X	[253]
cine MRI (low res.)	human	BCS	RO-UKF	A	—	[254]

### 3.3. Model parametrization

Model parametrization is typically achieved by optimization, attempting to match some target data with model result(s) using the *goodness of fit* gauged by an objective function. What is deemed a successful parametrization depends on the purpose. For some applications, the goal is simply to find a set of parameters that yield an acceptably low objective function, providing a model which appropriately matches data. However, when the parameters themselves are the target of an analysis, then the process of model parametrization should also consider the uniqueness and identifiability of parameters.

Model parameter uniqueness and identifiability depend on the model, the data and the objective function [244]. Important for parameter uniqueness is that a model be *structurally* and *practically* identifiable. Structural identifiability ensures that given endless, error-free data, one could determine model parameters uniquely. In contrast, practical identifiability ensures that parameters can be determined uniquely based on the type of data available in experiments. Demonstrating practical identifiability is often straightforward for simple models, but becomes increasingly challenging as models become more complex. Even if a model demonstrates practical identifiability, experimental or clinical data can introduce problems. Noise and bias artefacts in data can reduce parameter identifiability and make model predictions unreliable. Adding to the challenge is *model fidelity*, or whether the model is sufficiently rich to represent that data. Model simplicity can lead to a poor match between the simulation and data and potential non-uniqueness in parameters. In contrast, complex models may rectify issues with model fidelity but at the expense of parameter identifiability. The objective function also plays an important role, acting as the yardstick indicating whether model and data agree. If this objective is too relaxed, it may be possible for multiple parameter sets to be valid minima despite parameters being, theoretically, uniquely identifiable. Considering these factors is critical for studies wishing to use model parameters themselves.

Parameter identification using *in vivo* imaging data of the beating heart has challenged many researchers, especially when using the exponential-type strain energy density functions to model the passive mechanical behaviour of myocardium. The lack of 3D kinematic measurements, or of certain modes of deformation during normal *in vivo* motion, may result in poor practical identifiability. For example, during diastolic filling, the motion information captured during *in vivo* imaging may not adequately span into the nonlinear range of the stress–strain (or pressure–volume) curve to enable the underlying material parameters to be fully characterized [245]. Schmid *et al*. [259] conducted simulation studies to examine the suitability of five strain-based constitutive models and determined the orthotropic exponential constitutive model developed by Costa *et al*. [49] to best represent different shear deformations [260]. However, several studies have reported strong correlations between the material constants [244,246,247].

### 3.4. Data assimilation

The previous sections illustrated the wealth of models and data available for simulating the heart. These models can be calibrated (parametrized) based on patient data to produce patient-specific simulations. In mathematical terms, the process becomes an inverse problem, whereby data that is generally a potential output of the model system is used to help retrieve specific model inputs (e.g. unknown initial conditions, physical parameters, etc). Cardiac models, by their nature, are dynamical systems of partial differential equations (PDEs) and their integration with data to solve inverse problems falls into the general class of methods referred to as data assimilation techniques. Historically, data assimilation arose in geophysics in the 1970s [261], but nowadays has reached new fields of science [262]. From its inception, data assimilation relates to numerous theoretical fields: statistical estimation, optimization, control and observation theory, modelling and numerical analysis, as well as data processing. In cases where the complete solution, for example the entire motion of the heart, is known, some have suggested direct use of the data to determine unknown parameters by the direct use of these quantities within the governing PDE [263]. More often, however, data assimilation aims to reconstruct the trajectory of an observed PDE system from a time sequence of heterogeneous measurements. In data assimilation, there are two classes of methods: *variational* and *sequential* [262].

*Variational methods* seek to minimize a least-squares criterion combining a comparison term between the actual data and the simulation, with additional regularization terms accounting for the confidence in the model. The comparison-term or data-fitting-term is usually called similarity/discrepancy measure, whereas the model confidence terms are called *a priori*. This is known as the variational approach as reflected in the popular 4D-Var method [264]. The most effective minimization strategies compute the criterion gradient through an adjoint model integration corresponding to the dynamical model constraint under which the criterion is minimized. Then, the minimization problem is classically solved by a gradient descent algorithm, involving numerous iterations of the combination of the model and adjoint dynamics. Gradient-free approaches can also be used, simplifying the use of variational methods in complex codes, but involving still more evaluations of the functional [253,265].

*Sequential methods* can be inspired from the point of view of feedback control theory. The key concept is to define an *observer* (also referred to as estimator in the stochastic context) that uses the data as a control to track the actual trajectory, and concurrently retrieve the unknown parameters. This so-called sequential approach gives a coupled model–data system solved similarly to a usual PDE-based model, with a comparable computational cost. Indeed, this approach can provide significantly improved efficiency assuming the feedback is designed specifically for the system at hand. Two main classes of feedback have been developed: (1) reduced-order Kalman-based feedbacks (or filters) which cumulate the advantage of their *genericity* with a reduced computation cost for PDE-based models [266,267]; (2) Luenberger feedbacks where the filter is specifically tuned by analysing the dissipative properties of the physical system described by the models [268,269]. Note that these two classes can be used concurrently to define, for instance, joint state and parameters estimators [266] where the Luenberger filter is devoted to the stabilization of the state errors (initial conditions, modelling errors) and the reduced-order Kalman-like feedback handles the parameter identification. We can also combine these two feedbacks for coupled physical systems, for instance in cardiac electromechanics where a Luenberger feedback is used in the mechanics and a reduced-order feedback is employed on the electrophysiology [270]. Use of established data assimilation techniques for cardiac mechanics requires some special considerations. The most fundamental one is to establish the observability of the system, namely the amount of information that can be retrieved from the data at hand. This question is vast and must be addressed using sensitivity analysis and uncertainty quantification.

#### 3.4.1. Methodological issues in cardiac data assimilation

Use of established data assimilation techniques for cardiac mechanics requires some special considerations. Heart models present a major difficulty in that they are strongly nonlinear, with phase changes throughout the cardiac cycle. These changes in the physical nature of the heart can introduce challenges in the use and convergence of data assimilation methods [250]. In this respect, the (sequential) methods that rely on *particles* for the model sensitivity computations have proved to improve robustness [247,249,250]. Other constraints on the system (e.g. tissue incompressibility discussed in §2.4) or the parameters that often lack direct observations must also be handled appropriately. Moreover, it is often beneficial to add physical constraints (such as positivity for material stiffness constants) by adjusting the physical system or through a change of variables [249,250].

Another difficulty arises due to the data at hand. In cardiac mechanics, the first source of data is image sequences providing motion through the cardiac cycle. The information contained in images is very different from the classical model outputs, which makes the definition of similarity measures challenging. In essence, the model computes displacements with respect to a reference configuration. In contrast, the images show the deformed domain over time. With some imaging modalities, such as tagged MRI or 3D ECHO, image processing techniques allow one to reconstruct a measured 3D displacement sequence more directly comparable to the model displacements [244,246]. However, for standard MRI or CT sequences, we must define a discrepancy measure between the deformed model domain and the observed shape. In this respect, the data assimilation community may benefit from the data fitting definitions already well developed in the image processing and registration community [271].

## 4. Bringing translational cardiac modelling to the clinic

**Figure 4.**
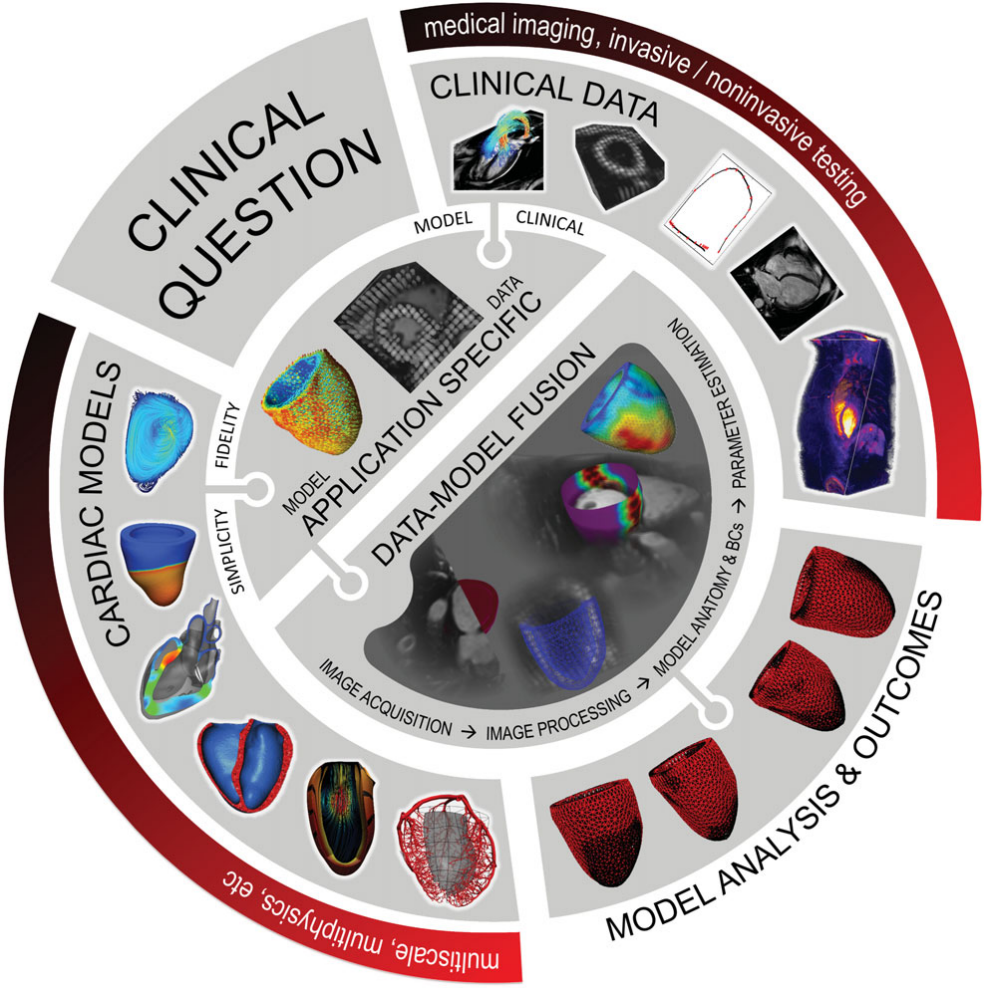
TCM pathway, illustrating the formative steps of model-based analysis. The driver for TCM efforts starts with the clinical question, informing the selection of an application-specific model that brings together the appropriate data and model components. Data–model fusion is then required, personalizing the model with sufficient data (either patient-specific or population average data) to address the clinical need. Once formulated, modelling can be executed and used to generate specific clinically relevant outcomes, informing diagnosis, treatment optimization or treatment planning.

The advancements made in modelling, imaging, image processing and data assimilation provide an impressively diverse range of tools and data. Extending these developments beyond academic and research realms and into the hospital requires careful consideration of specific clinical questions and the requirements of the end-user. The specific clinical application and desired outcome, in turn, guide the selection of required models and data, influencing the necessary processing and assimilation tools, theoretical considerations, etc. (figure 4). The pathways for TCM to make an impact clinically are numerous. In this section, we highlight some of these active TCM efforts.

### 4.1. Device assessment

Device-based treatment and therapy are playing an increasingly important role in the clinic [272]. Considering the significant time and investment required to bring medical devices to market, thorough vetting of a device's design is critical before it enters into clinical trial. Modelling, with encouragement from regulatory agencies [273], has played a significant role in device evaluation, particularly in the testing of mechanical- and tissue-based heart valves [134] which were simulated in idealized geometries to examine performance, damage and fatigue.

**Figure 5.**
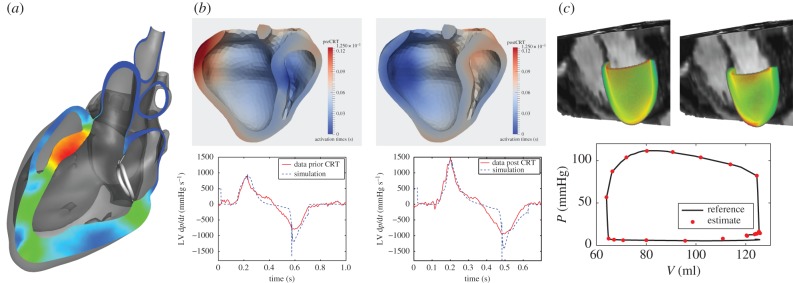
Example applications bringing TCM to the clinic. (*a*) Evaluation of mitral annuloplasty device using a four chamber electromechanical heart model, assessing the degree to which the device improves mitral valve regurgitation [274]. (*b*) Examination of biventricular CRT, using an electromechanical model tuned to baseline data to predict therapy response of left ventricular d*p*/d*t* [99]. (*c*) Left ventricular mechanics model parametrization using CINE, 3D tagged and 4D PC MRI providing estimates of tissue properties through the cardiac cycle [244,252].

Increasingly, modelling is being used to consider how a device interacts with the mechanics of the heart itself. Figure 5*a* illustrates the use of an electromechanical model used to access the efficacy of a mitral valve annuloplasty device that aims to reduce mitral regurgitation. FSI models have been applied to assess left ventricular assist devices [100,101,161], examining how alterations in device settings influence myocardial unloading as well as the potential for LV thrombus formation. Electromechanical modelling has been used to assess the AdjuCor (http://www.adjucor.com/home.html) extravascular ventricular assist device, whereby pneumatic cushions are used to improve ventricular stroke volume while unloading the heart [275]. Biomechanical modelling has also been used for trans-catheter aortic valve replacement, using an anatomically accurate aortic model to simulate valve deployment [276].

These examples illustrate the potential of TCM to provide a platform for the rapid evaluation of medical devices. Further advancement of computational techniques [277–279] enhances the ability of models to resolve fine details influential in the operation of devices. In this context, models rely on anatomically accurate (as opposed to patient-specific) geometries and literature-based values to effectively represent the heart. Here, the purpose is a model with representative patient physiology, providing a means for evaluating efficacy and suggesting design modifications. This shift in focus dramatically simplifies data–model fusion and provides a more straightforward platform for exploiting complex cardiac models.

### 4.2. Therapy planning

Another avenue for TCM is through therapy planning, where models are used to predict the outcomes of different therapies. A prime example is Cardiac Resynchronization Therapy (CRT), where the placement of leads to excite the heart are currently evaluated during device implantation to maximize the rate of left ventricular pressure at early systole [280]. The peak left ventricle pressure time derivative (max LV d*p*/d*t*) is a standard clinical measure of a short-term effect of CRT. Electromechanical models tuned to pre-therapy imaging data were shown to accurately predict the effect of biventricular pacing (figure 5*b*) [99]. Modelling results also provided insight into therapy, suggesting that length-dependence (Frank–Starling mechanism) might be a key factor in treatment efficacy [39]. Both the studies used non-invasive MRI data as well as invasive electrophysiological data. Use of these tools to optimize treatment prior to surgery requires reducing the dependence on invasive data, a direction currently being explored.

Modelling is also being pursued as a way to evaluate pharmacological interventions, particularly when multiple drugs may be combined to deliver an optimal therapy. The syndrome of heart failure would once more be a typical example [281]. Significant efforts are also spent on electrophysiological side, searching for potential cellular proteins that could be targeted for drug development [282–284]. Inherently, this effort requires multiscale models to effectively examine the cascade of a drug operating on a subcellular target to a functional at the organ level. Extending these models beyond the development stage towards patient-specific planning introduces challenges and will likely require significant investment into the identification of key alterations required for personalization.

While many therapy models focus on the acute response, consideration of growth and remodelling effects is extremely attractive in predicting the long-term viability of therapy. These models are increasingly important as many therapies, such as CRT, are known to result in reverse remodelling and thus fundamentally change the responsiveness of the heart to treatment over time. Growth and remodelling could also play a role in therapy planning, where often the decision of whether or not to treat a patient is made based on the likely deterioration in a patient's condition [285]. Using these modelling approaches could provide much more reliable predictors of disease progression, enabling appropriate staging of therapy. Key to this development is the appropriate identification of remodelling mechanisms through either animal experiments or clinical studies where invasive tissue samples can be collected.

### 4.3. Biomarkers and diagnosis

Beyond addressing specific therapies, TCM has been proposed as a novel path for patient assessment and potential diagnosis through the use of model-based biomarkers. Advances in cardiac imaging and catheterization techniques have resulted in a wealth of clinical data contributing to the design of numerous biomarkers for cardiac dysfunction. Leveraging these data using patient-specific cardiac models provides a useful tool to better understand cardiac dysfunction on an individualized basis [99,244,246]. While estimation of quantities, such as stress and work, from direct measurements is not currently feasible, these quantities can be directly estimated using personalized models, providing a wealth of information that could be exploited to stratify patients.

Further, the data assimilation and model personalization processes require the tuning of model parameters. Often these parameters are quantities of interest, such as myocardial tissue stiffness or contractility [249,252,254] (figure 5*c*). Unlike other clinical indices that only reflect global chamber performance, myocardial mechanical properties provide tissue-specific biomarkers elucidating potential muscle tissue fibrosis, scar, reduced contractility and/or delayed relaxation. *In vivo* measurement of tissue mechanical properties is not readily available; therefore, estimates derived from model-based analysis integrating available personalized clinical data can offer more insight into the mechanisms and potential stage of disease. Recently, quantifying myocardial tissue properties has become the focus of much research to better understand the causes of ventricular dysfunction in several cardiac diseases such as myocardial infarction, heart failure [255], heart failure with preserved/reduced ejection fraction and hypertensive heart disease. *In vivo* estimates of tissue mechanical properties combined with other subject-specific measurements of cardiac function may assist with more effective stratification of different types of heart failure. Moreover, use of model-based biomarkers that can be rigorously tested against current clinical standards [286] would enable the design of mechanism-specific treatment strategies.

## 5. Realizing translational potential

### 5.1. The clinical question and data/model selection

With the wealth of available models and data, it becomes evermore critical that TCM is guided by the requisite outcome required to address a clinical question (figure 4). Identification of the problem and the intended outcome enables the appropriate identification of a model and set of data that ensures accuracy and robustness. This interplay introduces a delicate balance between the clinical question, available data, model fidelity and the final outcome desired from the model. A model must be rich enough in function to address the question of interest. It may be richer than necessary so long as the additional complexity does not yield uncertainty (or non-uniqueness) in the outcome. In contrast, simplicity often yields robustness but this can come at the expense of accuracy. As a consequence, realizing the potential of TCM requires careful consideration of the key factors important for addressing the clinical question at hand.

The examples of models applied into clinical problems from §4 fall into two general groups: direct translation to clinics and an indirect TCM employing the models in the development of new techniques (e.g. devices or drugs). Both application types require a very tight collaboration between the modelling team and end-customer, whether it be clinicians or commercial partners. Building bridges between these historically disjunct cultures is beginning at centres around the globe, as clinicians, industrial partners and modellers work more closely together to address relevant problems. Such a tight collaboration is necessary to ensure TCM addresses core needs and end-customers know what can be reliably derived from model-based outcomes. A successful example of translational modelling is HeartFlow (https://www.heartflow.com), illustrates the need for inter-disciplinary teamwork well, being co-founded by both a cardiovascular surgeon and engineer.

Clinical data, in particular medical image-based data, remain a necessary component to the progression of TCM. Improving imaging and image processing techniques will have a significant influence on the accuracy and robustness of model-based outcomes. This integration must begin at a much more fundamental level, making image acquisition and processing account for modelling needs and adapting models to robustly use these outputs. Imaging modalities (such as elastography [235–237] and *in vivo* diffusion tensor imaging [234,238]) present new opportunities to further engage modelling and imaging towards clinical outcomes, providing more direct measures of tissue properties and structure. However, the combination of imaging and modelling must also remain cognizant of clinical constraints, balancing outcomes with the complexity of patient assessment, cost and timescales. Nowadays, TCM often relies on the most advanced data available, which are often far from those acquired through standard care. In a research context, efforts to exploit the wealth of available data are essential to explore the potential for TCM outcomes. However, for clinical practice to shift towards such complex exams, the value of a model must be demonstrably better than the current standard of care. Using routine clinical data may limit the information for TCM, but would significantly improve uptake by facilitating the organization of multi-centre clinical studies. In this context, information-rich databases of standard data (e.g. UK Biobank; http://www.ukbiobank.ac.uk) will likely prove invaluable for guiding TCM efforts.

The selection of a cardiac model needs to balance the clinical question and available data. Importantly, the model-based outcome must address the question, capitalize on the available data, be robust and minimize uncertainties. Even the most efficient data assimilation methods become significantly more challenging and computationally intensive as the number of personalized parameters grows or the computational problem itself increases in demand. As a consequence, simplified models are often being used to parametrize components of more complex models; as was the case in [100] where a solid mechanics–Windkessel model was used to tune model parameters prior to simulating the full FSI-Windkessel model. Moreover, very complex models encompassing the widest range of physiological knowledge through integration many smaller models are not necessarily more predictive or reliable. The trade-off between model fidelity and simplicity remains application-specific. A simplistic (or complex) model may provide valuable outcomes in one application but be completely flawed in another.

While multiphysics models are necessary for addressing some phenomena in the heart, such integrated models often come with a greater demand for personalization and increased computational complexity. Examining the added value of such a model becomes increasingly important in TCM, where clinical timescales are short and computational resources in the majority of clinical centres are limited. While computational techniques and hardware continue to improve at rapid pace, it is likely that the most significant impact for multiphysics models will occur in the assessment of devices. By using population average models, the need for personalization would be eliminated, while still providing a valuable *in silico* environment for testing.

Despite broad support for multiscale modelling and simulations of cardiac growth and remodelling, the difficulty of model validation, mainly caused by the lack of appropriate experimental and clinical data, remains a point of concern [287]. Determining the genesis of physiological changes due to drug-based interventions and validating currently proposed hypotheses on governing principles is critical to the long-term use of modelling for guiding therapy. One important step is to extract the important cardiac modifications due to remodelling in order to guide modelling. Recent developments in statistical shape models for longitudinal analysis can help extracting this information [285,288]. However, exploiting the full potential of these techniques will require longitudinal animal experiments, collecting imaging and biopsy data to quantify alterations across scales.

### 5.2. Application-specific models and data–model fusion

Once an application-specific model and suitable type of data are selected, data collection and data–model fusion present a number of practical challenges that must be addressed to maximize translational potential. Imaging protocols, though typically standardized, are often adjusted to the patient's status and may be negatively influenced by the compliance of the patient or skill of the operator. These factors can significantly degrade image quality or precision, reducing the efficacy of TCM. Mitigating these factors through more straightforward imaging protocols, better identification of image quality measures, or using data redundancy to identify the confidence in image-derived quantities would greatly improve reliability of imaging data. Another core challenge is the streamlining and automation of image processing and data assimilation pipelines. While many tools exist in the research environment, few can be robustly applied by clinicians across many datasets. Moreover, manual intervention is often necessary, introducing uncertainty which must be quantified to practically ensure the quality of results.

As model-based tools continue to advance, efforts have been initiated to cross validate and benchmark methods. In the domain of image processing, algorithms to track motion from tagged MRI were systematically evaluated and compared with manually segmented results [289], showing comparable accuracy among the methods tested. Comparison of different cardiac mechanical models was recently conducted on the same extensive pre-clinical dataset (STACOM 2014 challenge; http://stacom.cardiacatlas.org) to evaluate predictive capacity and compare model produced outcomes [290]. While quantities such as displacements were well-predicted, strong variations were observed in myocardial stress predictions across models. Recent work in FSI benchmarking has also been organized, using 3D printing and measured materials to test the predictive accuracy of these methods (http://cheart.co.uk/other-projects/fsi-benchmark/). Benchmarking and validation will be increasingly necessary for TCM to convince the clinical community and the regulatory agencies of the validity and robustness of application-specific models.

### 5.3. Model analysis and outcomes

For TCM to realize its potential, it is crucial that simulation results are mapped into appropriate quantities that can guide clinical decision-making. Many of the modelling results target standard clinical metrics (e.g. FFR, max d*p*/d*t*), enabling a more straightforward pathway for TCM to make a clinical impact. Novel biomarkers will likely arise from TCM (e.g. myocardial stiffness or contractility); however, beyond being robust and reliable, these quantities must be demonstrated to provide diagnostic or prognostic value beyond the current clinical norm. Identification of these potential targets requires strong collaboration between clinicians and modellers, mixing practical hypotheses with deliverable model metrics to assess a patient's state or therapy plan.

While uptake of TCM into clinical care would take significant time and resource, the advent of large databases storing longitudinal data on patient treatment and outcomes provides a pathway to significantly accelerate translation of model-based outcomes. An alternative pathway for identifying TCM targets is through large-scale statistical methods. In this context, model-based outcomes could be used along with other measures typically embedded in *Big Data* approaches, examining potential correlations between derived model-based outcomes and specific clinical conditions or responses to therapy. As model-based outcomes integrate data and physical principles, these could provide essential metrics that are non-trivially related to typical clinical measures. Replicating these measures using statistical methods alone would likely require significantly larger amounts of data and increasingly complex, nonlinear regression techniques.

### 5.4. Uncertainty quantification

Model-based approaches need to provide a measure of confidence in their predictions. As measurement on living tissue is, by nature, sparse and noisy, there is a strong need to integrate all the sources of error in the process and quantify their impact on model outcomes. This requires an important shift from the current deterministic approaches to more probabilistic strategies, where uncertainties in input data are modelled to understand their impact on outcomes. The challenge of determining the impact of uncertainty permeates through the entire translational modelling pathway (figure 4). Uncertainty in model boundary conditions and anatomical construction stemming from data requires careful consideration of likely errors inherent in the data and processing pipeline. Similarly, examination of data assimilation techniques and the variation of model parameters to uncertainty in data must also be considered. This can create challenges both methodologically, as deriving stochastic models of such complex phenomena is non-trivial, and computationally, as such approaches are much more demanding. While comprehensive assessment of all uncertainties presents significant challenges, better clarification of model outcomes is mandatory to hone the focus of these efforts. Verification that all model parameters and quantities maintain a certain accuracy is an ideal, but far away goal. However, more immediate confidence may be obtained through demonstration that targeted outcomes are robust and reliable. Uncertainty quantification methods have started to be applied in the cardiac community [187,249,291–293] and these techniques will play an increasingly important role in TCM.

## 6. Conclusion

Addressing current clinical limitations in diagnosis, prognosis, treatment and therapy planning in heart and cardiovascular disease remains a significant translational goal driving cardiac research. In this paper, we reviewed modelling efforts aimed at addressing various physiological mechanisms influential for cardiac mechanics—spanning spatial scales and physical principles. The substantial growth in medical imaging and the techniques for leveraging this data for modelling were also reviewed. These parallel developments have opened a broad range of possibilities for bringing TCM into the clinic.
